# Modalities for Visualization of Cortical Bone Remodeling: The Past, Present, and Future

**DOI:** 10.3389/fendo.2015.00122

**Published:** 2015-08-11

**Authors:** Kimberly D. Harrison, David M. L. Cooper

**Affiliations:** ^1^Department of Anatomy and Cell Biology, University of Saskatchewan, Saskatoon, SK, Canada

**Keywords:** basic multicellular unit, bone, remodeling, micro-CT, synchrotron

## Abstract

Bone’s ability to respond to load-related phenomena and repair microdamage is achieved through the remodeling process, which renews bone by activating groups of cells known as basic multicellular units (BMUs). The products of BMUs, secondary osteons, have been extensively studied via classic two-dimensional techniques, which have provided a wealth of information on how histomorphology relates to skeletal structure and function. Remodeling is critical in maintaining healthy bone tissue; however, in osteoporotic bone, imbalanced resorption results in increased bone fragility and fracture. With increasing life expectancy, such degenerative bone diseases are a growing concern. The three-dimensional (3D) morphology of BMUs and their correlation to function, however, are not well-characterized and little is known about the specific mechanisms that initiate and regulate their activity within cortical bone. We believe a key limitation has been the lack of 3D information about BMU morphology and activity. Thus, this paper reviews methodologies for 3D investigation of cortical bone remodeling and, specifically, structures associated with BMU activity (resorption spaces) and the structures they create (secondary osteons), spanning from histology to modern *ex vivo* imaging modalities, culminating with the growing potential of *in vivo* imaging. This collection of papers focuses on the theme of “putting the ‘*why*’ back into bone architecture.” Remodeling is one of two mechanisms “*how*” bone structure is dynamically modified and thus an improved 3D understanding of this fundamental process is crucial to ultimately understanding the “*why*.”

## Introduction

Bone tissue is three-dimensionally (3D) complex in structure and undergoes continual dynamic change. Despite its rigid structure, it is remarkable in its ability to adapt in response to mechanical stimuli associated with loading and to microdamage endured throughout life. Since Clopton Havers’ (1) description of “Haversian” canals and iconic works describing microscopic bone structure/function relationships (2, 3), it has been well appreciated that bone renews itself via the turnover of tissue, which we have come to know as “remodeling” (4). Remodeling is critical for maintaining healthy bone tissue; however, it can also lead to age-related bone loss through an imbalance between osteoclastic (bone resorption) and osteoblastic (bone formation) activity. A progressive deficit in bone formation leads to enlarged osteonal canals and thus increased cortical porosity (5–7). Ultimately, this contributes to bone’s fragility which is characteristic of osteoporosis (7, 8). Related fractures are significant events in the lives of those afflicted and are frequently associated with serious complications and even mortality. With increasing life expectancy, osteoporosis and other degenerative diseases of bone are a growing concern for health care systems worldwide (9). As such, study of the spatio-temporal regulation of remodeling is a topic of great significance within bone biology with the potential to impact many lives.

**Figure 1:**
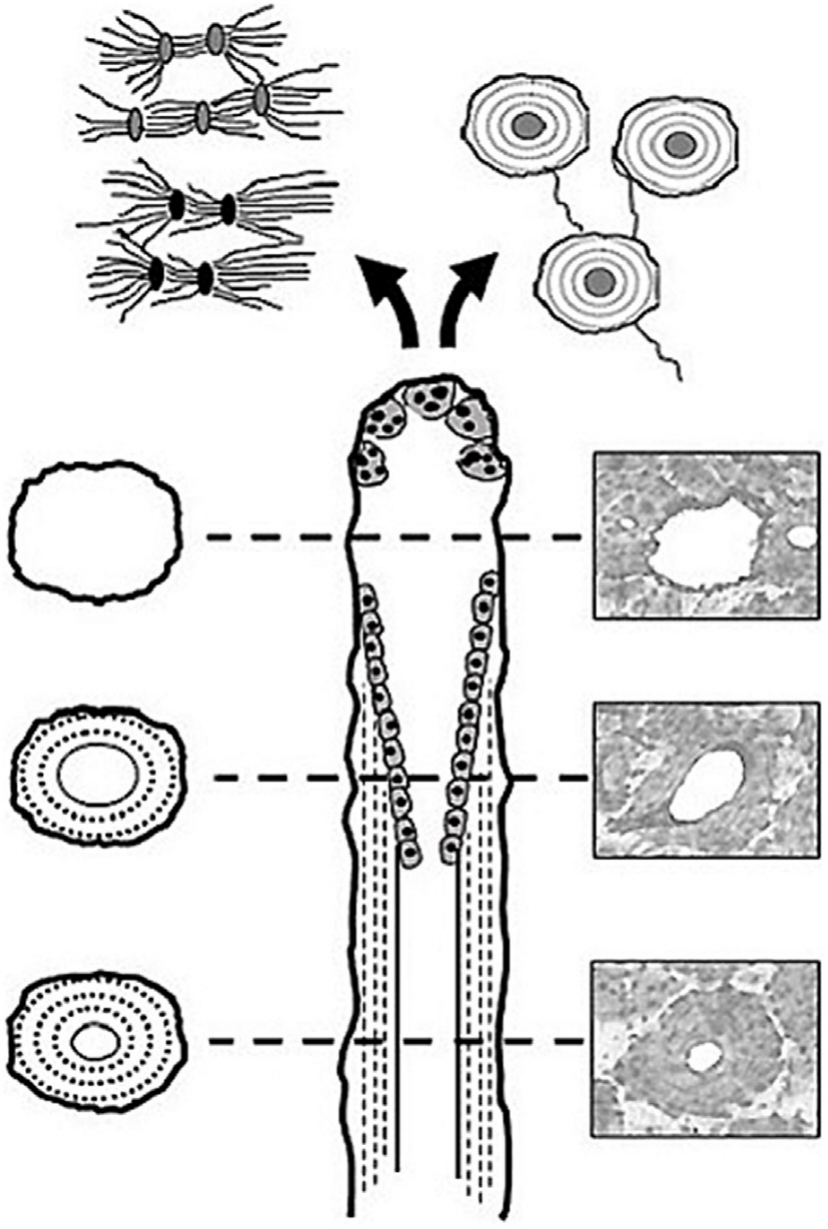
**Illustration of a BMU showing the classic ‘cutting’ and ‘closing’ cone morphology**. Osteoclasts located in the BMU’s cutting cone are attracted to areas of damaged bone indicated by the microcrack [based on Ref. (26)], as well as change in the canalicular network (black lacunae indicative of osteocyte apoptosis) caused by mechanical stimuli such as cyclic loading (based on Ref. (24)).

First described by Frost (10), basic multicellular units (BMUs) are the cellular groups responsible for carrying out the remodeling process. In cortical bone, this is achieved through the localized resorption of a cylindrical space (osteoclastic “cutting cone”) followed by concentric infilling of new tissue (osteoblastic “closing cone”) (Figure 1). The resulting structure is referred to as a secondary osteon (synonymous with “*Haversian system*”). As BMUs organizationally lie between the level of the cell and that of the tissue, Frost referred to them as “intermediary” (11). Despite decades of study, our understanding of the intermediary organization of bone remains rudimentary. BMUs are temporary collections of cells brought together to turnover a discrete packet of bone. Their course through bone tissue, their “behavior,” is challenging to directly probe, and thus much of our understanding has been inferred from osteon morphology. The orientations of secondary osteons appear to reflect principal stresses (12–16), and thus it has been hypothesized that the progression of BMUs is influenced by mechanical stimuli. This is not surprising as the two-dimensional (2D) geometry of secondary osteons has been linked to the function of the bones in which they are found (17, 18) and resultant mechanical strains (19). Additional examples include intra-element regional (i.e., anterior, posterior, lateral, medial) variation in osteon morphology (20, 21) and a relation between osteon size and weight observed in humans (22). To explain the link between mechanics and BMU orientation, computational (*in silico*) modeling has looked to stimuli such as localized strain (23) and strain-related fluid flow (24) around cutting cones. Such *in silico* models continue to become increasingly sophisticated, extending into the realm of simulation (25). All models, however, have relied upon highly idealized BMU morphology, and it is unclear how compatible their findings are with the more complex 3D morphologies which have been reported.

Another hypothesis regarding BMU regulation holds that their activities are spatially “targeted” (27) to remove damage manifested as microcracks (16, 28–30). While debate remains over the degree to which remodeling is targeted vs. untargeted (26), if targeting occurs, even for a portion of remodeling events, there must be mechanisms which actively steer BMUs toward damaged areas (Figure 1). This has been envisioned as attraction toward an “effective damage removal area,” which provides the means by which the osteoclasts of a BMU are drawn toward microdamage (15). Although it has been demonstrated that remodeling-related resorption spaces are associated with microcracks (31), active steering has yet to be empirically demonstrated. It is possible that BMUs are simply initiated in damaged areas. Further, other stimuli clearly play a role, and thus a clear dichotomy between targeted vs. non-targeted views of BMU regulation is problematic and they need not necessarily be mutually exclusive (27). The classical view of remodeling has always been envisioned as a multi-functional role – including mechanical and physiological functions (26) such as calcium homeostasis. Efforts to disentangle the multi-faceted regulation of remodeling would be greatly aided by more and better 3D data regarding BMUs and related structures.

In sum, the capacity to directly test hypotheses related to regulation of BMU activity and/or the validation of *in silico* models is limited by a general lack of 3D data. Indeed, the activity of BMUs has largely been inferred from 2D observation of the secondary osteons they create. Our appreciation of the 3D structure of secondary osteons is similarly limited, and those data which are available (discussed below) consistently hint at greater structural complexity than commonly appreciated. Improving our 3D understanding of cortical bone microarchitecture would, thus, enhance our understanding of the remodeling process. As such, the objective of this paper is to provide an overview of methodologies for 3D investigation of cortical bone remodeling and, specifically, structures associated with BMU activity (resorption spaces) and the structures they create (secondary osteons). This review will survey a range of approaches spanning from histology to modern *ex vivo* imaging modalities, culminating with the growing potential of *in vivo* imaging. As such, it will span past, present, and emerging approaches. This collection of papers focuses on the theme of “putting the “*why*” back into bone architecture.” Remodeling is one of two mechanisms “*how*” bone structure is dynamically modified, and thus an improved 3D understanding of this fundamental process is crucial to ultimately understanding the “*why*.”

## Past: Histological Approaches

Long before the advent of modern 3D imaging modalities [e.g., confocal microscopy, scanning electron microscopy, and microcomputed tomography (micro-CT)], traditional light microscopy yielded a wealth of information about bone microarchitecture. Early applications of light microscopy revealed remodeling-related structures including resorption spaces, mature osteons, and the canals within these osteons. The study of ground sections led to the first hypotheses pertaining to functional significance. Among the earliest of observations was a link between the extent of remodeling and age. Amprino and Bairati’s (32) study of compact bone from humans and animals was one of the first to show an association between age and osteon population density (i.e. the number of osteons/mm^2^). Why this occurs, either through targeted replacement or through the accumulation of stochastic events, remains a central debate in the field. It is not surprising then than much remains to be learned from histology and that histology, in various forms, remains a mainstay of bone biology to this day. There are, however, limitations to extrapolating 3D structure from sections – a leap which requires idealization and assumption (33). Indeed, histomorphology can vary significantly due to differences in sampling location. The formation of an osteon by a BMU is an excellent example. Remodeling can create a new osteon anywhere along a bone’s diaphysis and the resorption space in which it forms can extend over several millimeters (34). Stout et al. (35) found that sectioning osteons at different points along their lengths could result in the erroneous classification of different morphological “types.” They provided the example of a “dumbbell-shaped” osteon being explained as the product of sectioning through a branching event. Beyond morphology of individual osteons, the rate of remodeling can vary within a bone. Skedros et al. (18), for example, demonstrated that the number of osteons present in the forelimb bones of Rocky Mountain mule deer increased in a proximal to distal fashion. While larger fields of view (FOV) have become increasingly feasible through the use of tiling microscopes, FOV has been a significant limitation of microscopy. This is especially problematic for the measure of rate of remodeling – reflected by the measure “activation frequency,” which is defined as the number of new BMUs present in a particular unit of bone (10). Activation frequency can directly affect overall bone mass, because “remodeling space” temporarily reduces overall bone mass until it is subsequently filled in Ref. (36). Thus, a high remodeling rate can, in part, account for low mass. Activation frequency can be measured in either 2D sections (BMUs/mm^2^/unit time) (10) or 3D volumes (BMUs/mm^3^/unit time) based on the number of BMUs created per unit volume of cortex per unit time (37). The 3D extent of BMUs – their length or “range” – is a very difficult parameter to assess from histology, even from longitudinal sections. This parameter has the potential to impact the regional assessment of activation frequency with longer resorption spaces having increased potential to be detected in section. Cooper et al. (34) micro-CT analysis of 99 BMUs in human bone provided a measure of range varying between ~0.8 and 5.4 mm and most likely longer as they were limited to a 7 mm FOV and some remodeling events extended beyond. Keeping in mind the significant impact of sample site selection, activation frequency measures based on how *many* BMU-related resorption spaces are visible in sections acquired only millimeters apart from one another could be very different. Another problem with typical activation frequency measures is that, in section it can be difficult to differentiate BMU-related resorption spaces from other structures such as Volkmann’s canals (36). That being said histology-based analytical techniques are still the gold standard for visualization of bone microstructure. Indeed, histology remains the primary means of investigating the relation between remodeling and microdamage. When identifying microdamage, in the form of microcracks, it is critical to ensure one is detecting *in vivo* damage and not artifacts of processing – particularly when utilizing grinding-based approaches as they can produce artifacts within bone that resemble cracks induced *in vivo* (38, 39). Microcracks are generally observed in 2D sections (40, 41), and thus their overall morphology, and relation to remodeling events, can be difficult to ascertain. For example, comparison of microcrack morphological features, such as size and shape, viewed in 2D has been shown to exhibit differences, to the extent that individual cracks look like entirely distinct structures (41, 42). This was seen to be the case in a study by Voide et al. (43), which found certain microcracks appeared linear and confined in the cross-sectional plane, whereas viewed in the perpendicular plane, they appeared diffuse.

Serial sectioning can alleviate some of these issues associated with 2D histology. Multiple sections increase the amount of bone analyzed and provide direct insight into the 3D nature of microarchitecture. Cohen and Harris’ (44) analysis of serial decalcified sections from canine femora reported that osteons follow a spiral orientation around both the axis of the bone and axes of the osteons themselves. This seminal 3D study also reported that morphological characteristics vary in their distribution and this variation is linked to the specific locations within the bone. They observed that osteon cross-sectional area increased as they coursed distally throughout the bone and were also greater in the endosteal as opposed to subperisosteal regions (44), a finding suggestive of a mechanical influence on osteon morphology and thus BMU activity. Tappen’s (45) work with block section staining using silver nitrate revealed morphological characteristics of osteonal canals, lacunae, osteocytes, and canaliculi. Tappen (45) highlighted numerous features of osteons, and the remodeling process which included levels of stain uptake in bone can be used as an indicator of mineralization; BMU-related resorption spaces are continuous with canals; these spaces can tunnel in opposite directions from one another or in only one direction, and some unforeseen force(s) dictate osteon/resorption space diameter as evidenced by the areas of mineralized bone resorbed in active areas of remodeling.

Despite its advantages, serial sectioning is challenging and thus only a few significant attempts to investigate cortical bone microstructure by this approach have been published (35, 44–46). Notably, these few studies have not found consensus with respect to some general aspects of osteon morphology. For example, Stout et al. (35) digital analysis of Tappen’s (45) serial sections did not find evidence that osteons follow a spiral pattern around the axes of bone as suggested by Cohen and Harris (44). Rather, they found a complex pattern of branching osteons. Indeed, all 3D studies have revealed greater structural complexity than is frequently appreciated in the literature. While the literature is dominated by the “classical” BMU morphology presented in Figure 1, it should be noted that data indicating a complex diversity of remodeling-event types was reported in the mid 1960s by Johnson (4). We would argue that a lack of practical and efficient 3D techniques has contributed to the corresponding lack of information regarding BMU and osteon morphology. Beyond the difficult and tedious nature of existing serial-sectioning approaches, no small measure of luck is required to target these techniques to study individual BMUs and/or their related structures. Methodological difficulties ultimately led to the exploration of imaging-based approaches to the study of bone remodeling.

## Present: *Ex Vivo* Imaging

**Figure 2:**
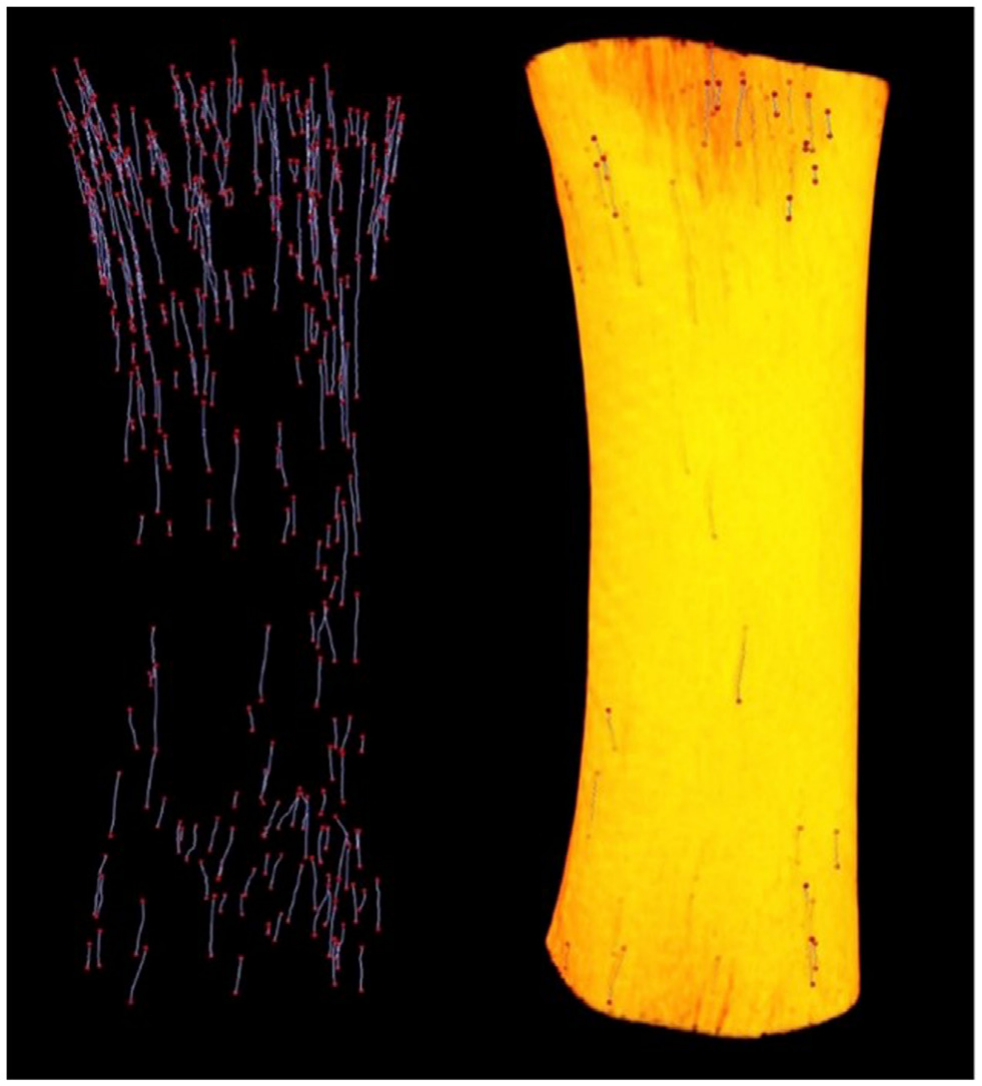
**Left: reconstructed micro-CT image of landmarked BMUs in a black bear metacarpal; right: 3D render of bone diaphysis superimposed over BMUs. Diaphysis length = 31.84 mm**.

Since its introduction by Feldkamp et al. (47), X-ray micro-CT has become the “gold standard” for the non-destructive 3D analysis of trabecular bone morphology. More recently, this technology has increasingly been applied to cortical bone to analyze the porous network of canals in 3D (6, 14, 34, 48–53). With respect to the study of remodeling, micro-CT represents an excellent approach for detection of BMU-related resorption spaces since their distinctive cutting cones generally stand out from the relatively smaller canals of completed osteons. Another key advantage of micro-CT is its ability to non-destructively survey a large volume of bone for active remodeling events. An excellent example of this is our group’s recent use of a laboratory micro-CT system (SkyScan 1172, Bruker, Belgium) to locate and trace BMUs in complete diaphyses of *Ursus americanus* (black bear) metacarpals and metatarsals without the requirement of sectioning (Figure 2) (54). In a more general sense, micro-CT can provide insight into remodeling through assessment of canal (and hence osteon) orientation (49) as well as the overall complexity of the canal network, which has been hypothesized to increase with cumulative remodeling activity (34, 48). The potential exists to examine osteon “steering” through assessment of localized canal orientation. Thus, the capabilities of micro-CT can alleviate or, at minimum, limit many issues associated with histological techniques for cortical bone: (1) interpolation of 3D structure from 2D sections is not needed as structures are observed directly and measured in 3D; (2) measures can be calculated in units of volume as opposed to area; (3) sample preparation is limited, eliminating structural alterations; (4) sample site location is less of an issue as visualization and analysis of larger volumes of interest – even entire bones – become feasible. These advantages are just beginning to be brought to bear on the *ex vivo* study of cortical remodeling, and micro-CT clearly has great potential to provide new insights through improved measures of morphology (including orientation, possible “steering,” and 3D range) and activation frequency.

**Figure 3:**
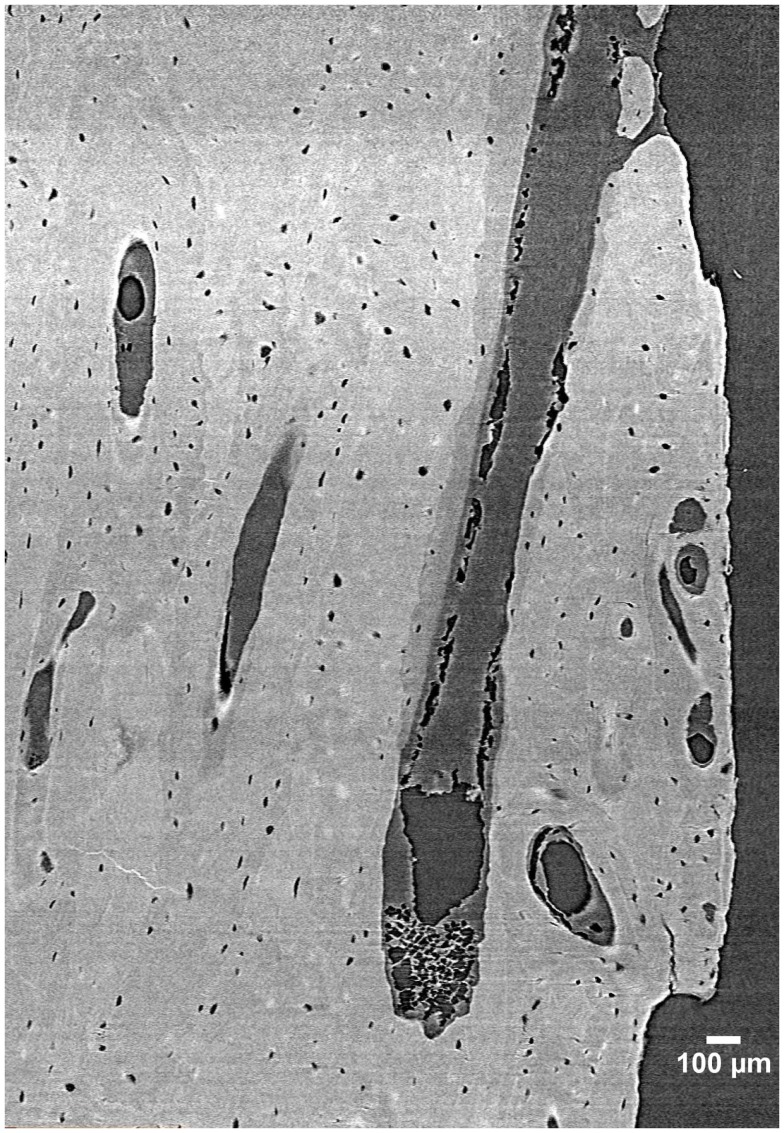
**3D reconstruction of a human femur section depicting BMUs and osteocyte lacunae acquired by SR micro-CT at a 1.47 µm resolution (58)**. Image provided by Dr. Yasmin Carter.

Despite its many benefits, micro-CT has several limitations. For cortical bone, the most notable is that while some delineation of osteon borders is possible from laboratory systems (55), this technology remains largely limited to detecting porosity at the vascular level and larger (e.g., osteonal canals and resorption spaces). Synchrotron radiation (SR) micro-CT, with its many advantages conveyed by greater X-ray flux, its monochromatic coherence, and high brilliance as a result of a small source size can be utilized to visualize smaller-scale structures including osteon borders (56, 57) and different osteon morphologies (57), osteocyte lacunae (58–60) (Figure 3), and even microcracks (42, 43, 61, 62).

A limitation shared by both micro-CT and SR micro-CT is that, in general terms, higher resolution comes at the cost of field of view. A further related limitation is that as resolution increases, so does the radiation dose. Improving resolution by a factor of 2 requires a dose increase by a factor of 16 to maintain image quality in micro-CT (63). This relation provides the practical limit on the resolution for *in vivo* X-ray based computed tomography. Thus, despite the fact that micro-CT imaging of trabecular bone microstructure in living animals is now commonplace (10–20 um voxel size), its application to the internal microstructure of cortical bone is not. An exception to this is the increasing use of high resolution-peripheral computed tomography (HR-pQCT) to analyze cortical porosity in humans, which will be discussed in the next section. The limited applications of micro-CT to the internal microstructure of cortical bone (e.g., osteonal canals) in animal models have all been *ex vivo* (64, 65). Working *ex vivo*, it is even possible to image microcracks utilizing barium sulfate as a contrast agent (66–68). With the vascular porosity of cortical bone beyond the reach of conventional *in vivo* micro-CT, it goes without saying that the resolutions required for microcrack imaging are not compatible with live animal imaging. Voide et al. (43) used SR micro-CT with a nominal resolution of 700 nm to visualize microcracks, *ex vivo*, in the femora of mice. Even if radiation dose was not an issue, holding a living animal still for sub-micron imaging would present a significant challenge in and of itself. Thus, while much can be learned about remodeling through *ex vivo* 3D imaging, *in vivo* detection and tracking of remodeling-related structures would represent a significant step forward with still greater potential to improve our understanding of this process. As will be discussed, there are specific advantages of synchrotron-based imaging which have created the potential for *in vivo* imaging of cortical porosity – opportunities that are just beginning to be explored and thus lie on the future horizon.

## Future: *In vivo* Imaging

A new generation of clinical research HR-pQCT scanners have given rise to a new opportunity for 3D, *in vivo*, characterization of human bone, both trabecular and cortical. With an isotropic voxel size of 82 μm, HR-pQCT has been at the forefront for micro-architectural analysis of the human appendicular skeleton (i.e., distal radius and tibia) (69, 71), as it is particularly advantageous for measuring cortical porosity (70). While HR-pQCT has been instrumental in studies concerned with osteoporosis induced change in microstructural cortical bone (71), its viability as a tool for targeting and tracking specific cortical structures, such as individual BMUs, is unclear due to resolution and other limitations associated with movement artifacts and the restriction to primary trabecular bone sites (i.e., wrists and ankles) (72). When one considers that the size of a single HR-pQCT voxel is on the same scale as the average canal diameter within human cortical bone, ~120 μm for pooled sexes (6), it becomes clear that this technology cannot resolve all osteonal canals. A recent study comparing HR-pQCT as a tool for measuring pore sizes against SR micro-CT showed that the accuracy of HR-pQCT was a factor of the size of the pores measured (70) – current resolution limits its ability to recognize small pores, thus introducing partial volume effects into analyses (72). That said, as BMU-related resorption spaces average 250 μm in diameter [e.g., the average diameter of an osteon in humans (22)], HR-pQCT may indeed hold the potential of tracking individual remodeling events in human cortical bone. Thus, while HR-pQCT represents a powerful new tool for the assessment of cortical porosity, it is limited to the largest of cortical pores located in the ultra-distal peripheral skeleton.

Human-based studies are clearly an important avenue for study, but more mechanistic studies will require controlled model systems. When considering animal models – particularly smaller animals – the punishing relation between resolution and radiation dose complicates *in vivo* imaging. The risks associated with high X-ray doses are not only harmful to a living subject in an acute sense, they include the potential to alter bone structure, leading to osteopenia, growth arrest, fracture, and malignancy (73) through altered osteoclast and osteoblast activity (74). For example, at a voxel size of 10.5 μm and a radiation dose of 845.9 mGy, longitudinal scans of the hind limb in mice acquired over a 5-week period resulted in decreased bone volume and increased trabecular separation (75). Even though SR micro-CT makes *in vivo* imaging of cortical porosity more plausible through higher resolution and shorter scan times, the limitations imposed by radiation dose are still a factor. As will be discussed, the monochromatic (single X-ray energy) capabilities of synchrotrons enable alternative methods of developing contrast beyond absorption. These so-called “phase contrast” techniques are opening the door to new possibilities for *in vivo* imaging.

**Figure 4:**
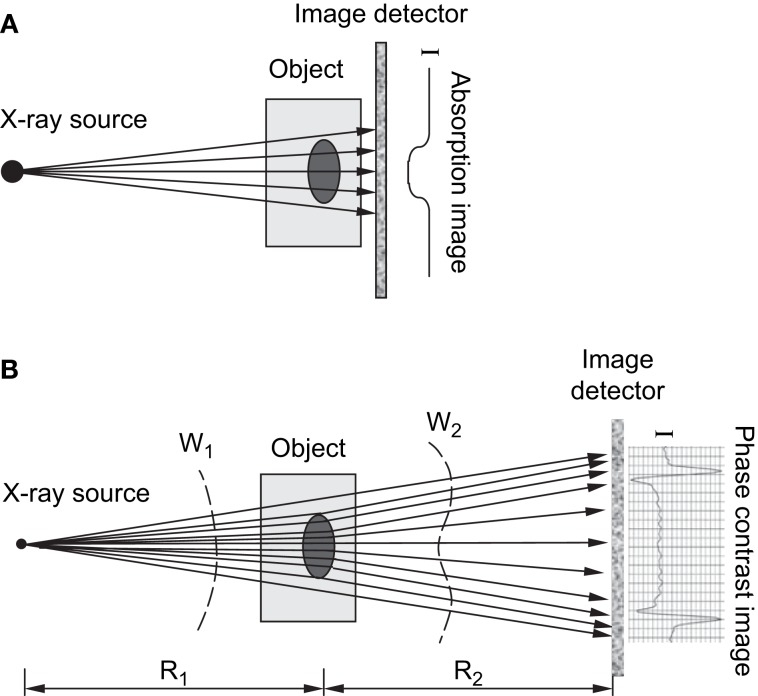
**(A)** Schematic of attenuation based X-ray imaging where images are produced based on the degree of absorption relative to an object’s internal structure. (B) Schematic of in-line phase-contrast imaging based on an object’s refractive properties. As X-rays target an object at different angles, variations in the object’s internal structure will refract the X-rays and cause a shift in the light wave as it propagates through and increasing the object-to-detector distance will produce a contrast image (76). Reprint permission granted by the publisher.

Several forms of phase contrast imaging, including in-line phase contrast, diffraction-enhanced imaging, and interferometer-based imaging, have been increasingly explored for their potential utilization in biomedical applications. An excellent overview of these methodologies is provided by Zhou and Brahme (76). Unlike conventional radiography, where images are based on the differences in X-ray absorption related to an object’s internal structure, phase contrast images contain information related to the refractive index of an object because the images produced are derived from the pattern of interference created from diffracted and undiffracted waves (77). Differences in refractive index of the sample’s internal structure will refract or bend the X-ray wavefront as they pass through the target and these differences can be used to generate contrast (76). For in-line phase contrast imaging – the simplest of these techniques to implement – detection of changes in X-ray refractive indices within a sample can be optimized by altering the distance between the object and the detector (76, 78) (Figure 4). Simple implementation and enhanced detection of internal features have made the fusion of SR computed tomography and phase contrast imaging particularly advantageous for visualization of microscopic bone structures such as osteocyte lacunae (58, 79, 80), and even nanoscopic structures such as the lacuno-canalicular network (59). Phase contrast is not dependent on X-ray attenuation and thus higher energies, where attenuation is lower, can be employed (76). This creates the possibility of higher resolution with equal or reduced dose compared with attenuation-based imaging.

Our group recently tested the feasibility of in-line phase contrast SR micro-CT for imaging the cortical porosity in the forelimb of rats with radiation doses comparable to those commonly employed *in vivo* for imaging trabecular microarchitecture (78). Since its’ first implementation (81), numerous *in vivo* SR micro-CT protocols varying in both dose and image resolution have been utilized to visualize the microarchitecture of bone in small animal models (78, 82–84). Currently, there is no consensus for what is considered to be a safe dose for live animal *in vivo* imaging. Voxel sizes in the 10–15 μm range are typical for laboratory micro-CT with dose rates of 0.4, 0.338, and 0.939 Gy, respectively (85–87). However, at a voxel size of 11.7 μm, Matsumoto et al.’s (83) *in vivo* scans of the knee joint in living mice involving 5 Gy showed no signs of radiation sickness. Based upon this context, our goal was to create an *in vivo* imaging protocol involving a dose as close as possible to those used routinely in conventional micro-CT and no higher than 5 Gy.

**Figure 5:**
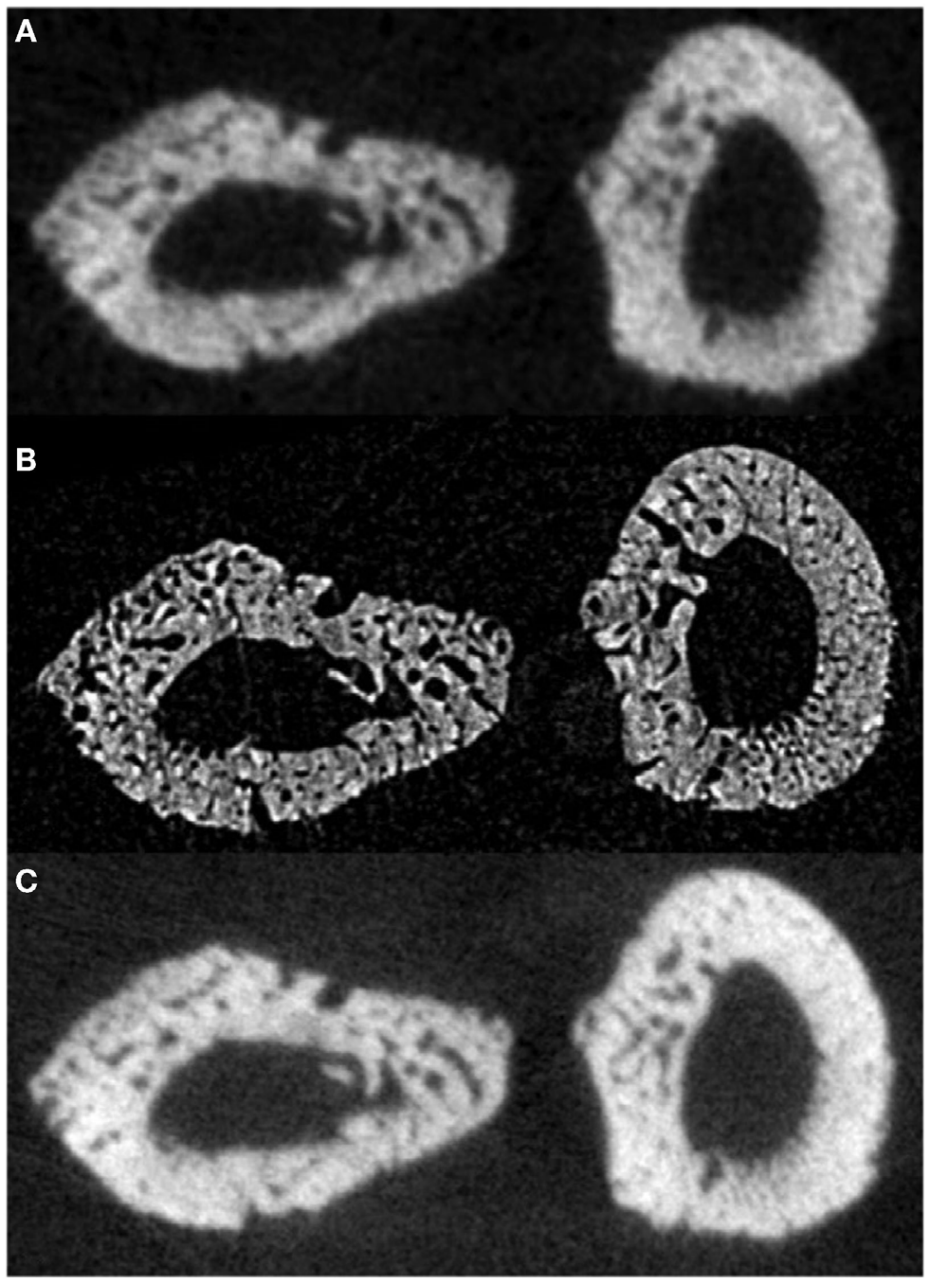
**Reconstructed slices of rat forelimbs depicting visualization of cortical porosity based on the imaging system used: (A)** *in vivo* laboratory SkyScan 1176 micro-CT (18 μm, 1.2–1.5 Gy dose), (B) *in vivo* synchrotron micro-CT slice measured using the C4742-56-12HR camera (11.8 μm, 2.53 Gy dose), (C) *in vivo* laboratory SkyScan 1176 (9 μm, 11.7–18.2 Gy dose) (78). Reprint permission granted by the publisher.

**Figure 6:**
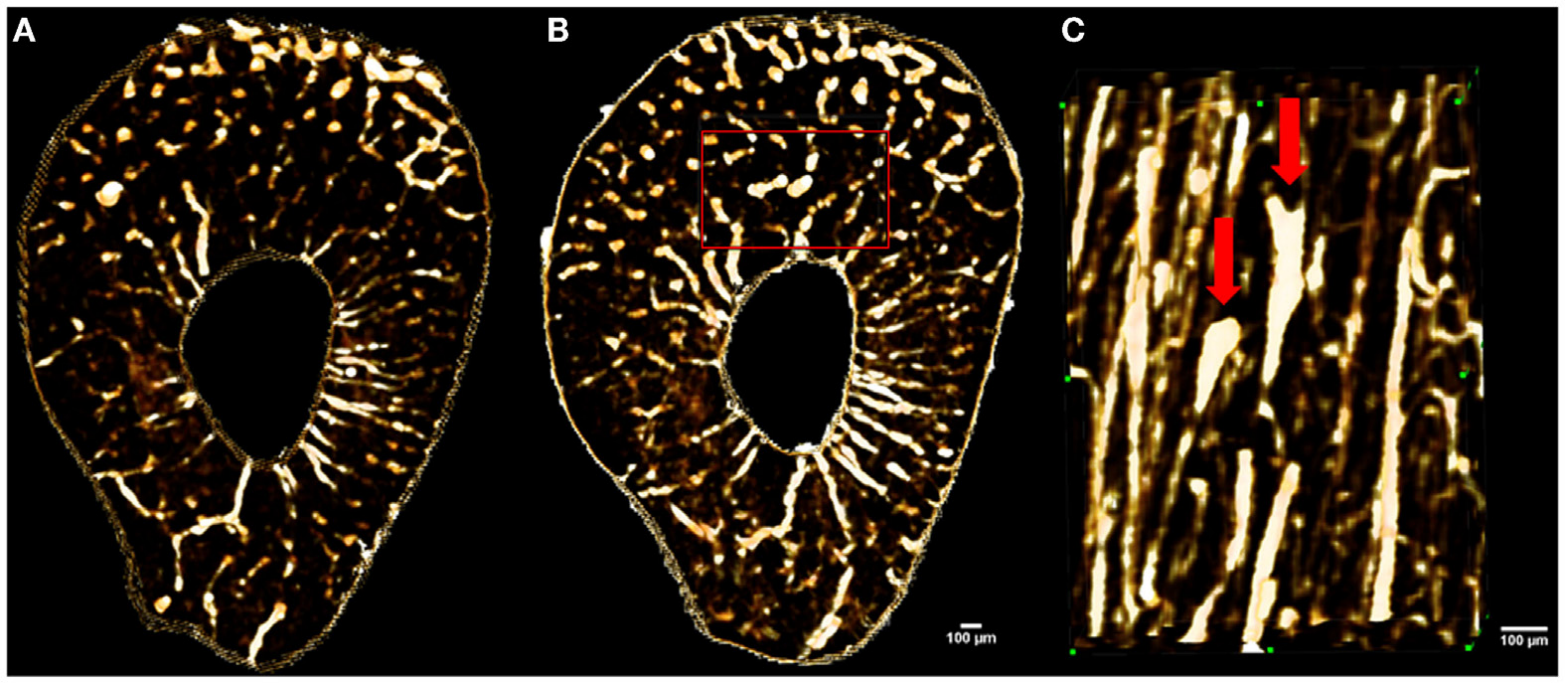
**Images showing *in vivo* matched scans of a rat forelimb acquired with SR micro-CT (11.8 μm, 2.53 Gy)**. Scan **(B)** was carried out two weeks after scan **(A)** on the same rat’s forelimb. Image **(C)** is an enlarged section of image **(B)** (red rectangle) displaying new remodeling events (red arrows).

In a proof-of-principle study, *ex vivo* data collected at the biomedical imaging and therapy (BMIT) facility of the Canadian light source (CLS) synchrotron were compared against laboratory micro-CT protocols and their related doses. Studying rat forelimbs, it was found that SR provided superior detection of cortical pores without a substantial increase in dose (11.8 μm voxels, 2.53 Gy) beyond that used in the laboratory systems (18 μm voxels, 1.2–1.5 Gy; 9 μm voxels, 11.7–18.2 Gy) (78) (Figure 5). For in-line phase SR micro-CT, the optimal target-to-detector distance for our configuration was found to be 0.9 m. Subsequent to the *ex vivo* trials, the first *in vivo* trial was performed (78) and the first longitudinal studies employing this protocol are under way – providing encouraging results with respect to tracking individual remodeling events (Figure 6).

Synchrotron radiation micro-CT, taking advantage of in-line phase contrast, has brought about a new opportunity for *in vivo* imaging to directly test causative hypotheses relating to cortical bone remodeling; however, it too is not without its limitations. Access to synchrotrons is inherently limited due to the relative scarcity of such facilities and, in particular, those with biomedical-focused facilities capable of imaging live animals. Even with the high resolution afforded by SR micro-CT and the ability to minimize dose through use of in-line phase contrast imaging, some structures such as osteon borders and microcracks are not observable, *in vivo*. Indeed, such structures remain challenging to image *ex vivo*, requiring very high-resolution systems. Movement artifacts are problematic as the difficulty in holding the animal perfectly still increases along with resolution. The smallest of the standard animal models (e.g., mice and rats) have the further drawback of not exhibiting much, if any, natural cortical remodeling – although it can be induced by experimental means [e.g., fatigue loading in the rat (31)]. Notably, this imaging approach has the potential to be extended to larger animal models including those such as the rabbit, which exhibit remodeling under normal conditions. Larger cortical canals in such animals could also relax the resolution needs and, concomitantly, reduce radiation dose.

## Conclusion

The remodeling process, carried out by the activity of BMUs, is of great interest to the bone biology community. This process represents the primary means of skeletal change after maturity and lies at the root of many chronic bone diseases, including osteoporosis. Visualization of cortical bone microarchitecture and, specifically, the remodeling process have progressed from 2D histological analysis through *ex vivo* 3D imaging and now to *in vivo* 3D analysis. This progression has paralleled but lagged behind visualization of trabecular bone microarchitecture due to the smaller scale of the target features and the need for high resolution which suffers the complication of increased radiation dose for X-ray based imaging. An important consideration is the caveat that while this progression involves a decreasing level of sample destruction/invasiveness, there is a trade off in terms of the structures one can visualize. The imaging approaches are best suited to detection of porosity. Thus, for some applications, histology (serial or otherwise) remains the most powerful or possibly only approach available. That said, there is a great potential to combine the strengths of these approaches – fusing 2D and 3D imaging to maximize the information available. This will enable targeted histology – directed by imaging data. Looking to the near future, we believe that this approach will see application in direct testing of hypotheses related to the regulation of BMU activity – including questions related to steering/orientation, the role of microcracks, and the relation to other stimuli, including possibly cellular signals. Such data will, in turn, prove invaluable for validating *in silico* models – an area of increasing focus in bone biology. Ultimately, we believe the novel insights possible through 3D data will shed significant new light on the “how” of bone aging, adaptation, and disease. Understanding the “how” is critical to understand the “why.”

## Conflict of Interest Statement

The authors declare that the research was conducted in the absence of any commercial or financial relationships that could be construed as a potential conflict of interest.
